# Time-resolved biophysical approaches to nucleocytoplasmic transport

**DOI:** 10.1016/j.csbj.2017.03.005

**Published:** 2017-04-04

**Authors:** Francesco Cardarelli

**Affiliations:** Center for Nanotechnology Innovation @NEST, Istituto Italiano di Tecnologia, Piazza San Silvestro 12, 56127 Pisa, Italy

**Keywords:** Fluorescence recovery after photobleaching, Single particle tracking, Fluorescence correlation spectroscopy, Diffusion, Transport, GFP, Nuclear pore complex, Live cell, Confocal microscopy

## Abstract

Molecules are continuously shuttling across the nuclear envelope barrier that separates the nucleus from the cytoplasm. Instead of being just a barrier to diffusion, the nuclear envelope is rather a complex filter that provides eukaryotes with an elaborate spatiotemporal regulation of fundamental molecular processes, such as gene expression and protein translation. Given the highly dynamic nature of nucleocytoplasmic transport, during the past few decades large efforts were devoted to the development and application of time resolved, fluorescence-based, biophysical methods to capture the details of molecular motion across the nuclear envelope. These methods are here divided into three major *classes*, according to the differences in the way they report on the molecular process of nucleocytoplasmic transport. In detail, the first class encompasses those methods based on the perturbation of the fluorescence signal, also known as ensemble-averaging methods, which average the behavior of many molecules (across many pores). The second class comprises those methods based on the localization of *single* fluorescently-labelled molecules and tracking of their position in space and time, potentially across *single* pores. Finally, the third class encompasses methods based on the statistical analysis of spontaneous fluorescence fluctuations out of the equilibrium or stationary state of the system. In this case, the behavior of *single* molecules is probed in presence of many similarly-labelled molecules, without dwelling on any of them. Here these three classes, with their respective pros and cons as well as their main applications to nucleocytoplasmic shuttling will be briefly reviewed and discussed.

## 1 Introduction

In eukaryotic cells, the cytoplasm and the nucleus are spatially separated by a double membrane, the nuclear envelope (NE). Embedded in the NE are the nuclear pore complexes (NPCs), which allow the passage of ions and molecules across the NE and, at the same time, regulate the exchange of larger molecules, such as RNAs, proteins, or ribonucleoprotein (RNP) particles between nucleus and cytoplasm [1].

The overall shape of the pore is known since pioneering studies, among others, were conducted yeast by electron microscopy (EM) [2] and on *Dictyostelium discoideum* by cryo-electron tomography (cryo-ET) [3]: the pore is a channel-like structure of about 40–90 nm in length and 40–75 nm in width, showing an asymmetric structure with flexible protein filaments extending out from the pore (approximately 50 nm) into the cytoplasmic environment, and an open basket-like structure extending to about 75 nm into the nucleus. More recently, mainly thanks to the straightforward combination of EM and ET with mass spectrometry (MS) analysis, structural modelling, and X-ray crystallography, our knowledge about the finest structural details of the NPC is enormously increasing (for more details see Refs. [3], [4], [5], [6], [7]). Collectively, the achievements brought by structural studies promise to open new perspectives for our understanding of the molecular mechanisms underlying NPC function in normal and altered conditions [8].

At the molecular level, the whole NPC consists of about 30 different polypeptides designated nucleoporins (Nups), with a very controlled stoichiometry, and a total mass of ~ 125 MDa [9], [10], [11]. Most of the Nups lack a fixed secondary structure but rather contain domains rich in phenylalanine-glycine (FG) repeats [12] which are very flexible. These FG-Nups are generally located within the central channel of the NPC, forming a selective barrier that inhibits the efficient translocation of large molecules (> 40 kDa) unless they are chaperoned by transport receptors [13], such as Importin β (Impβ). Impβ, one of the major transport receptors, recognizes cargo molecules in the cytoplasm and forms a transport complex either directly or indirectly (i.e. through Importin α, Impα) [14]. Cargo-receptor complexes are able to interact with Nups at the cytoplasmic filaments or at the peripheries of the central pore [15]. From here, the cargo-receptor complex is transferred to the FG-repeat domain of nucleoporins in the center of the NPC (e.g. Nup153 [16]), where the FG-Nups offer a functional redundancy of binding sites for Impβ [17], [18]. How nucleoporin-Impβ interaction drives NPC-passage is, however, not a trivial problem, and several models address this issue (for a detailed review refer to [19]). In spite of their variety, these models differ mainly in the physical arrangement and mobility (static vs. dynamic) of the FG-domains within the NPC. Whatever the organization of the FG-Nups in the NPC, the transport process is terminated by the intervention of Ran guanosine triphosphate (RanGTP), which dissociates Impβ from the FG-Nups at the level of the nuclear basket and causes the release of the cargo molecule into the nucleus. The newly formed Impβ-RanGTP complex is selectively transferred into the cytoplasm to initiate a new round of transport.

**Fig. 1:**
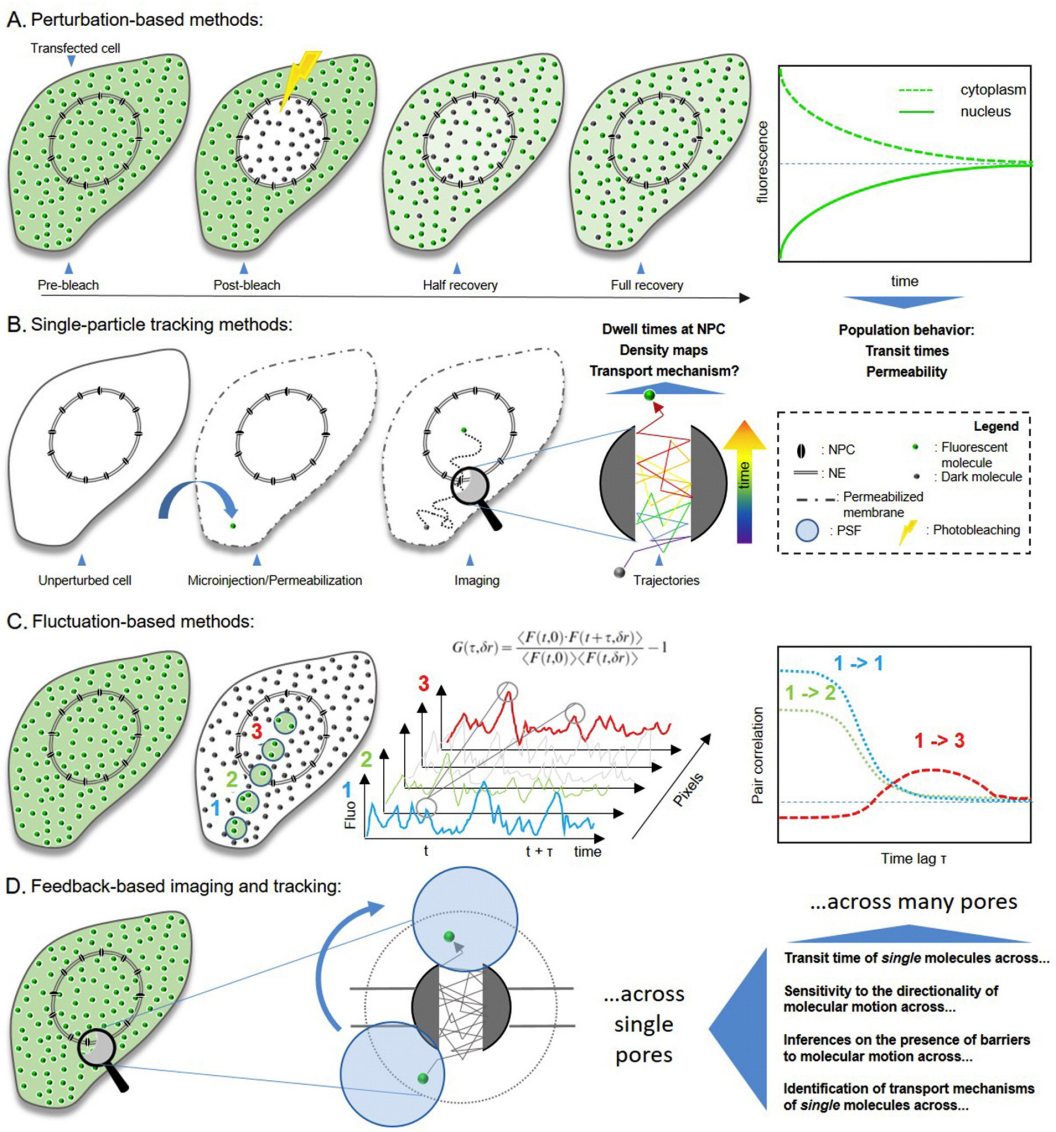
Schematic representation of the major classes of time-resolved biophysical approaches to nucleocytoplasmic transport. A) Perturbation-based methods. A schematic representation of the FRAP method is reported, with the nucleus of a cell being phobleached to then follow the recovery of fluorescence due to the exchange of ‘dark’ and ‘green’ molecules across the NE. A typical plot of exponential fluorescence recovery in the nucleus (and concomitant decrease in the cytoplasm) is reported. From such a measurement, under proper modelling of the process under study, the dynamic behavior of a population of molecules can be extracted, in terms of characteristic time of fluorescence recovery, immobile/mobile fraction of molecules, etc. B) Localization-based techniques. Typically, the molecule of interest must be properly purified, labelled, and introduced into the sample by microinjection or permeabilization procedures. At this point, single-molecule imaging can be performed, provided that the label yields the required amount of photons to allow localization with the desired precision. Under optimal conditions, trajectories of single molecules transported across the pore can be described (as schematically represented here). From trajectories, residency times at the pore and/or density maps of single-molecule localizations can be extracted. C) Fluctuation-based techniques rely on the rapid acquisition of fluorescence signal fluctuations from a system (e.g. a transfected cell as in the example here) left at equilibrium or steady state (no large perturbation is introduced). Spatiotemporal analysis of such fluctuations (e.g. by the pair correlation function reported here) provide sensitivity to single molecules in presence of many similarly labelled molecules, large amount of information in a single measurement and compatibility with the use of relatively dim molecules (e.g. GFPs) in live, unperturbed cells. For instance, by the pCF algorithm, average transit times of single molecules across (many) pores can be measured and inferences about the nature of the pore as a barrier to molecular motion can be drawn. D) Feedback-based methods. In this case, the observation volume defined by the PSF is rapidly orbiting around the object to be tracked (the pore in this case), with a response time of few milliseconds and a location precision in the nanometer range. In other words, standard analytical tools (e.g. fluctuation analysis) can be brought onto the reference system of a *single* pore to follow the translocation of *single* molecules with great precision and a time resolution that is faster than the motion of the overall NPC.

Given the highly dynamic nature of the overall process, a variety of time resolved biophysical strategies were applied to nucleocytoplasmic transport of molecules (summarized in Fig. 1). They can be roughly divided into three major classes, according to their differential capability to report on the behavior of molecular transport events across the NPC gateway.

The first comprises perturbation-based approaches, such as Fluorescence Recovery After Photobleaching (FRAP). By these methods, the characteristic time of molecular transport across the entire NE can be measured by averaging the behavior of many molecules, across many pores. A peculiarity of these methods is that they rely on the change of the optical properties within the sample only, whereas the characteristic dynamics and function of the molecules under study are not altered. Originally conceived by Peters in 1974 [20], this technique owes much of its relevance to the discovery and development of auto-fluorescent proteins (FPs) [21], a class of genetically-encoded fluorescent molecules derived from sea organisms such as jellyfish or corals. FP-based FRAP was successfully applied to many scientific fields, including biophysics and biomedicine, at cellular and subcellular level [22], [23], [24], [25], also taking advantage from the recent development of microscopy setups that allow high-resolution imaging on living cells [26].

The second class comprises localization-based single particle tracking (SPT) methods. By these approaches, the trajectories of single molecules of interest are measured. By SPT, the transit times and interactions at the level of isolated NPCs of several transport receptors and model cargo molecules were successfully probed. Contrary to other approaches, SPT experiments typically require complex experimental procedures: for instance, the molecule of interest must be purified, properly labelled (without affecting its functionality), and introduced into the cell by microinjection or cell permeabilization. Also SPT measurements inherently require bright and isolated particles that must localized and tracked over time many times in order to acquire reasonable statistics.

Finally, the third class encompasses spatiotemporal fluorescence correlation spectroscopy (FCS)-based approaches, which afford single molecule ‘sensitivity’ in presence of many similarly labelled molecules and in live, unperturbed cells. The recently developed spatial extension of FCS, named pair correlation function (pCF) approach, was proven to be particularly suited to study the shuttling of molecules across the NE [27] and other sub-cellular structures (e.g. chromatin territories [28], [29], [30]). This approach builds on the dual-foci FCS concept [31] and combines FCS and SPT potentialities into a new method in which the time needed for each molecule to be found in a given point in space that is different from the position at time zero can be extracted [32]. If a barrier to diffusion is present, a longer time will be needed for the same molecule to be found at a position across the barrier, as already demonstrated in the case of molecular transport across the NE [27].

In the following, the detailed contribution of these three classes of time-resolved methods to our understanding of nucleocytoplasmic transport of molecules will be summarized and discussed. A glimpse into the future directions in the field will be also provided.

## 2 Many Molecules Through Many Pores: Perturbation-based Approaches

Thanks both to the recent advancements in the optical microscopy technologies at our disposal and to the development of new fluorescent labels, several perturbation-based approaches were developed, including photobleaching methods (e.g. FRAP, see below), photoactivation (when nonfluorescent labels become fluorescent after illumination at a selected wavelength, e.g. by using the photoactivable GFP, or PA-GFP [33]), photoswitching (when fluorescence, upon excitation at a certain wavelength, can be switched on or off by light in a reversible manner, e.g. by using the DRONPA [34] or E222Q FP variants [35]) and photophotoconversion (when irradiation of labels at a selected wavelength induces a shift of their fluorescence spectrum toward longer wavelengths, e.g. by using the Dendra [36] and mEos [37] proteins). Irrespective of the method, after the photo-perturbation event, what is typically measured is how the fluorescence distribution relaxes toward the steady state. Among these strategies, FRAP is by far the most popular one and is widely used as a microscopy protocol suited to study the mobility of molecules and particles [21]. In a typical FRAP experiment (Fig. 1A), a short and intense light pulse is applied to irreversibly photobleach the fluorescent molecules in a selected region of the sample (e.g. the nucleus, as in Fig. 1A). After photobleaching, the “dark” molecules gradually transfer out of the photobleached area, while at the same time unbleached molecules enter it from the surroundings. This exchange leads to a fluorescence recovery within the photobleached area (and to the concomitant decrease of fluorescence in the surroundings) that can be monitored by low-intensity excitation (schematic plot in Fig. 1A). Analysis of the recovery curves by means of a suitable biophysical model yields insight into the dynamic behavior of the molecule under study. Among the dynamical cases amenable to FRAP analysis, a particularly interesting situation occurs when diffusion takes place between two (or more) compartments separated by a permeable membrane, a situation often encountered in biological systems where membrane compartmentalization is at the basis of life. Molecular diffusion through the NE falls into this class.

Concerning the available literature, in a pioneering work by Wei and colleagues [38], real-time imaging and FRAP were used to examine the nucleocytoplasmic shuttling of 27-kDa EGFP molecules in single live cells. It was found that EGFP diffuses bi-directionally through the pore, across the NE. The ∼ 100-fold slowing down of GFP diffusion at the NE barrier compared to free diffusion within the nucleus or the cytoplasm was interpreted as due to the reduced size of the NPC channel available for diffusion. The authors did not report any significant block of EGFP diffusion by depletion of perinuclear Ca^2 +^ stores. Also, EGFP bi-directional shuttling showed no variation with the cell cycle. These results, obtained on an inert tracer (i.e. GFP), have been considered as a reference for the study of passive diffusion of other molecules through the NPC. In fact, GFP is nowadays used as an indispensable benchmark in any attempt to identify the NPC translocation mechanism of endogenous proteins.

For instance, Sunn and co-workers, based on the GFP reference, were able to show for the first time that the vitamin D receptor B1 (VDRB1) exploits a serum-dependent, active nuclear transport process, while no active nuclear export mechanism was found for the same protein [39]. In a similar way, FRAP was successfully used to examine the nucleocytoplasmic transport of several other proteins or molecules including virus-derives nuclear localization signals (NLS) such as Tat peptide and the NLS from simian virus 40 (SV40) large tumor antigen (T-ag), members of the Importin family such as Imp13, and transcription factors such as STATs (signal transducers and activators of transcription) cancer regulatory proteins such as the parathyroid hormone related protein (PTHrP) signaling molecule or pRB (retinoblastoma protein) tumor suppressor [40], [41], [42], [43], [44], [45], [46], [47], [48], [49], [50].

Worthy of note, at this point, is the use of reversibly photoswitchable fluorescent proteins (RSFPs) (mentioned above in this section). In contrast to standard FRAP or photoactivation strategies, RSFPs enable the reversible optical highlighting of specific pools of molecules and thus the repeated measurements of protein dynamic behavior, definitely increasing the amount of information that can be retrieved from a single measurement. In the last 15 years, many of these FP variants have been proposed and new cell imaging applications discovered that exploit their properties [51]. In the context of nucleocytoplasmic shuttling, it is worth mentioning the pioneering study by Ando and colleagues, conducted on a DRONPA-labelled variant of Erk protein [34]. Exploiting photoswitching, the authors could identify Erk responses, in terms of translocation in and out of nucleus, in one cell under different, consecutive, stimuli (e.g. with and w/o EGF [34]). Concerning photoconvertible FPs, on the other hand, it is interesting to note that they enable to simultaneously detect both non-photoconverted and photoconverted subpopulations of labelled molecules, as showed by Chudakov and colleagues by studying Dendra2 redistribution across the nuclear envelope of living HeLa cells [52].

The methods described so far, being inherently ensemble-averaging strategies, do not afford information on single molecules but averages over many similarly-labelled molecules. This inherent limitation, however, does not prevent perturbation-based methods from providing quantitative information on the molecular (kinetic and thermodynamic) details of the transport process. For instance, by combining FRAP with the calibration of intracellular protein (i.e. GFP) concentration, Cardarelli and co-workers were able to quantitatively define the nuclear transport saturability by the estimate of the effective dissociation constant of the NLS-Importin complex in the actual cellular environment [47]. By this combined approach, the sub-saturation of the transport carriers (importins) by the NLS-cargo molecules was found to be a key factor regulating the overall nuclear import rates in living cells, in contrast to what expected based on the available in vitro data (conducted on purified components of the transport machinery). The same approach was then extended to the study of Nuclear Export Signal (NES), its affinity for the export machinery, and the maximum rate of NES-mediated transport at saturation of export carriers [53]. The measured quantities were found to be remarkably similar to those characteristic of active nuclear import. Our results also suggested that active export/import and active export/passive diffusion fluxes must be largely uncoupled, and that a mechanism of differential gating at the NPC level must exist.

## 3 Single Molecules Through Single Pores: Localization-based Approaches

As opposed to perturbation-based techniques, localization-based approaches potentially yield single molecule information, provided that the molecule of interest is properly labelled and introduced into the sample (e.g. a cell, through permeabilization/electroporation) at the desired concentration (steps schematically represented in Fig. 1B). In these conditions, localization-based methods can afford valuable information on structural and functional properties of the system under investigation [54]. A first distinguishing advantage of single molecule tracking experiments is that molecular processes do not need to be synchronized, as opposed to ensemble kinetic measurements, where it is usually challenging to obtain a population of molecules instantaneously triggered to start the process/reaction of interest. Also, single-molecule studies yield fluctuations and distributions of dynamical/kinetic parameters that are typically lost in ensemble-averaging experiments. Last, connected molecular reactions can be probed with no need to trap intermediates, as in the case of ensemble experiments.

Of particular interest for biological applications is the detection of single molecules by the use of high-sensitivity CCD camera systems in far-field optical microscopy setups [55]. The diffraction-limited image of single molecules is typically approximated through a two-dimensional Gaussian function interpolation. Although information on the shape of the sub-diffraction particle cannot be usually retrieved, its position can be determined with high precision. In particular, the localization accuracy depends on the signal/noise ratio and may reach a few nanometers under optimal experimental conditions [56], [57], thus allowing to carefully reconstruct the dynamic behavior (trajectories) of single molecules. Historically, single molecules studies have been widely applied to analyze the movement of molecules on membranes (e.g. single receptors and lipid molecules; for a review see [58]), but were concomitantly extended to the study of single-molecule mobility in the 3D interior of cells (for an extensive review see [59]). Concerning nucleocytoplasmic shuttling, single-molecule detection in a far-field optical setup was shown to yield significant insight into the molecular details of the transport process. For instance, by combining sensitive fluorescence microscopy with microinjection, Babcock and colleagues investigated the transport into the nucleus of influenza genes by real-time, live-cell 3D tracking of single viral ribonucleoproteins (vRNPs) [60]. The authors show unambiguously that vRNPs are transported within the cytoplasm and nucleus by passive diffusion, while they undergo binding to the NPC, with dissociation rate constants ranging from 1 to 100s. Also, the authors demonstrate how the expression of the protein M1 during late infection is able to downregulate the nuclear import of vRNP by directly inhibiting its binding to the NPCs^60^.

Concomitantly, in a series of studies conducted both in permeabilized and in microinjected cells, Kubitscheck and colleagues used far-field single-molecule microscopy to measure the distribution of binding sites and the dwell times at the NPC for a series of endogenous transport receptors with and without their respective transport substrates [61], [62]. Based on the obtained results the authors could argue for a molecular transport process with no significant interference between the nuclear import and export processes, in analogy with the FRAP-based results discussed above [53]. This evidence can be explained, in principle, by assuming that the pores are alternatively involved in export or import processes or that indeed two (structurally?) different pore species exist, one deputed to import and another to export. An additional scenario, increasingly supported by data, can be that the pore channel embeds independent (i.e. structurally separated) pathways for import and export (see below).

Interestingly, Yang and colleagues exploited a narrow-field confocal setup to further improve the S/N ratio of standard acquisitions [63], [64]. By this approach, they reached approximately 2 ms temporal resolution and 15-nm spatial resolution for a fixed molecule. The spatial resolution is obviously destined to decrease in the case of a mobile molecule, and inherently depends on how fast the molecule is moving. In this case, however, the S/N ratio was sufficiently high to directly measure the time a single molecule spends interacting with the NPC. The tracking algorithm reveals that molecules spend most of their transport time by randomly moving in the central channel of the NPC with escape from the channel being the major rate-limiting step of the process. Worthy of note, the same authors were able to reach a resolution of 9 nm and 400 μs in space and time, respectively, by introducing the SPEED method (Single-Point Edge-Excitation sub-Diffraction microscopy) [65], [66], [67]. With this improved resolution, the transport of several import/export/cargo molecules (e.g. Imp β1, mRNA) was probed in great detail in permeabilized cells. Transport pathways were described at molecular resolution and used to build 3D spatial density maps of interactions between the FG-rich central channel and the translocating molecules (for a review see [68]). In brief, the authors proved that Importins (with or without their cargoes) and mRNAs use distinct routes from that of the small passive molecules. At the same time, they found that active (at least in the case of import) and passive transport are not fully separated in space within the central channel and that the extent of their overlap shall depend on the size of the transiting molecule.

## 4 Single Molecules Through Multiple Pores: The Pair Correlation Approach

To tackle the characteristic limitations of SPT-based approaches discussed above (e.g. large labels, complex experimental procedures, high statistic required), fluorescence correlation spectroscopy techniques appear as ideal alternative strategies. Among others, the pair correlation function (pCF) method is particularly suited to study nucleocytoplasmic shuttling as it is able to measure the time needed for single molecules to migrate from one point to another within a living cell and in presence of many similarly labelled molecules (schematic representation of the method in Fig. 1C) [32]. Such a peculiar property of the pCF algorithm makes it suitable to provide a map of molecular transport times among arbitrary points in the cells and to detect the presence of barriers to diffusion with high (millisecond) temporal resolution and diffraction-limited spatial resolution (schematic plot in Fig. 1C). Concerning nucleocytoplasmic exchange, the pCF method has been applied to monitor the transport of a model protein substrate (NLS-GFP, as discussed in the previous sections) through NPCs in living cells [27]. Cardarelli and Gratton demonstrated that the pCF algorithm can easily detect the expected lengthening in the transit time of molecules if two positions across the nuclear barrier are correlated (maximum of correlation in the 100–500 ms range) as compared to two points at the same distance but within the cytoplasmic (or nuclear) compartments. On the average, NLS-GFP molecules are slowed down ∼ 40–100 times when they passively diffuse through the pore in the nucleus-to-cytoplasm direction (with respect to free intracellular diffusion), in keeping with several FRAP-based estimates [38], [47]. By contrast, if the same algorithm is applied in the cytoplasm-to-nucleus direction, the role of active, receptor-mediated nuclear import of NLS-GFP becomes evident. Accordingly, although passive-diffusion transit times are still measured, the pCF output becomes dominated by shorter transit times (1–40 ms range), characteristic of carrier-mediated transport, as measured by SPT techniques [61], [62], [63], [69]. Worthy of note, the NLS-GFP active transit times can be spatially resolved with respect to the distance from the NE. In particular, the pCF algorithm detects the fastest cytoplasm-to-nucleus transit times if the starting point is selected close to the NE barrier. This result correlates with the localization of endogenous Impα/β carriers, which are typically accumulated at the level of single pores [47]. Concerning this latter issue, it is worth mentioning that Bianchini et al. recently presented the combination of pCF with STimulated Emission Depletion (STED) to analyze diffusion below the diffraction limit [70]. The achievable spatial resolution by using overexpressed GFP tagged molecules was found to be around 110 nm in live cells (more than twofold improvement over conventional confocal imaging). STED-pCF highlighted how the intracellular environment close to the nuclear barrier affects the mobility of proteins which are actively imported into (or exported from) the nucleus. In fact, STED-pCF analysis unveiled the presence of local cytoplasmic and nucleoplasmic constrains to diffusion as well as the presence of slow diffusive component at distances up to approximately 1 μm from either sides of the NE. This latter slower component resembles that previously detected for transport complexes between cargo molecules and Importins. Remarkably, this level of accurate mapping of diffusion and its regulation is lost in conventional confocal imaging.

Overall, the broad distribution of transit times (around the maximum of correlation) is a characteristic of the pCF-based analysis of nuceocytoplasmic transport. This is due to the fact that, each time that two points are correlated across the NE, the contribution of all the *single* transport pathways allowed for molecules to travel from one location to the other are averaged together. Nonetheless, the single molecule sensitivity afforded by the pCF analysis is precluded to ensemble-averaging measurements, such as FRAP, and is particularly useful if many experimental conditions are to be quantitatively screened with high accuracy. As a bright example, Hinde and co-workers exploited pair correlation microscopy to show that polymeric nanoparticles with different shapes but identical surface chemistries moved across the various cellular barriers, including the NPC, at different rates, ultimately defining the site of drug release [71]. They measured how micelles, vesicles, rods and worms entered the cell and whether they escaped from endosomes and had access to the nucleus via the pore. Rods and worms, but not micelles and vesicles, entered the nucleus by passive diffusion. Their results demonstrate that drug delivery across the major cellular barrier, the NE, is important for doxorubicin efficiency and can be achieved with appropriately shaped nanoparticles.

Worthy of mention, recent conjugation of the pCF approach to Number&Brightness analysis opens up new perspectives for the study nucleocytoplasmic exchange in live cells [72]. In particular, Hinde and co-workers combined the pair correlation approach with molecular brightness analysis into a new method called pCOMB (pair correlation of molecular brightness). pCOMB filters the different oligomeric species diffusing within living cells and tracks their mobility based on transit time between two locations. Hinde and co-workers successfully used this approach to show the dependence of STAT3 (Signal Transducer and Activator of Transcription 3) intracellular mobility on its oligomeric state. They observed that, upon NPC translocation, STAT3 molecules in dimeric state must first bind to DNA to form STAT3 tetramers, which remain bound to DNA but acquire a different mobility. Cross-pair correlation (cpCOMB) analysis of the dimer-to-tetramer transition clearly shows that DNA accessibility is a key factor modulating STAT3 tetramer formation. Overall, the pCOMB approach was proved well suited for mapping the role of protein oligomerization in the regulation of transcription factor dynamics and function.

## 5 Summing Up *Pros* and *Cons*

It is worth stressing here that perturbation-, localization-, and fluctuation-based approaches are here presented and discussed not in general, but strictly related to their application to nucleocytoplasmic transport. Within such a peculiar biological context each of them shows selected distinguishing benefits or limitations (see Table 1). Ensemble averaging methods, for instance, are inherently limited in their ability to report on the molecular details of nucleocytoplasmic shuttling, as they can only provide bulk information on the overall process (averaging the contribution of all the molecules and all the pores in the cell). A typical perturbation-based experiment on nucleocytoplasmic transport is also inherently limited in the spatial resolution (S-res in Table 1), that is set by the extent of the photobleached area (and thus typically coincides with the size of the whole nucleus or cytoplasm), and in the temporal resolution (T-res in Table 1), that is typically set by the time needed to capture an image of the whole cell (i.e. from hundreds of milliseconds to seconds, except from line-scan FRAP in which few milliseconds can be technically reached [49]). On the other hand, however, perturbation-based measurements do not require complex sample-preparation procedures, can be performed on any standard optical setup and are thus accessible even to non-experts (for both data acquisition and data analysis). Localization-based approaches are, in theory, the preferred ones to describe molecular behavior, even in the case of nucleocytoplasmic transport, as they potentially provide information on single molecules (by means of the trajectories) moving across single pores. At the same time, however, localization is severely challenged by the movement of the molecule in a 3D environment and this in turn imposes a limit in the temporal resolution accessible (i.e. in this case the time required to collect enough photons for proper localization) and, consequently, in the final spatial resolution at which the trajectories of single molecules are described (please see Ref. 73 for further details). In the context of nucleocytoplasmic transport, these intrinsic limitations led to temporal and spatial resolutions in the order of hundreds of microseconds and few nanometers, respectively (e.g. see Ref. 65), which are actually sufficient to describe the dynamic behavior of molecules crossing the NPC. It is worth mentioning that, from a technical point of view, localization-based experiments rely on bright and isolated particles to be tracked many times in order to acquire enough statistics. Furthermore, most of these experiments require the molecule of interest to be properly purified and labelled (typically by using large particles (gold, quantum dots) that can modify the overall transport dynamics of the protein. Also, such experiments often demand for non-standard optical setups (e.g. Ref. 65) and expert users (for both data acquisition and data analysis). The spatiotemporal analysis of fluorescence fluctuations combines some of the technical advantages of perturbation-based approaches, such as the use of standard experimental procedures (e.g. use of GFP) and standard optical setups, with the intrinsic single-molecule sensitivity typical of localization-based techniques, but in this case in presence of many similarly labelled molecules. In the context of nucleocytoplasmic transport, fluctuation analysis on a standard line-scan acquisition provides a quantitative picture of molecular transit times (averaging the contribution of many single molecules across many pores) with adequate temporal resolution (typically hundreds of microseconds) and spatial resolution limited by diffraction [27]. Typically, fluctuation-based experiments are accessible even to non-experts, while data analysis requires specific skills. Worthy of note, in contrast to the other two classes of techniques, fluctuation spectroscopy offer higher multiplexing capabilities, in terms of its natural compatibility with additional tools, such as multi-channel detection (cross-correlation analysis), super-resolution methods (e.g. STED), additional fluctuation analysis (e.g. Number&Brightness), or feedback-based imaging strategies (see next section).

## 6 Future Directions

As mentioned in the introductory section, intense debate is still ongoing about the molecular details of nucleocytoplasmic transport and the nature of the selective gating imposed by the NPC. In particular, the structural and functional spatiotemporal organization of the FG-rich nucleoporins of the central channel (and their role in regulating molecular transport under native conditions) remains largely obscure. Available models are mainly based on simplified in vitro attempts to reconstruct the organization of selected components of the pore and are largely contradictory [16], [74]. To get valuable information in vivo, at least three main requisites are desirable: *i*) high spatial and temporal resolution, in order to properly describe the processes involved in nucleocytoplasmic transport; *ii*) single molecule sensitivity/resolution; *iii*) single pore resolution. In this regard, as already discussed, each of the presented strategies shows its own limitations. Perturbation-based methods, although informative, inherently fail to provide direct single-molecule and single-pore observations. SPT-based approaches are valuable as they potentially satisfy all the three requisites. Yet, it is not clear whether the experimental conditions required for SPT experiments (e.g. microinjection, permeabilization and use of large, bulky labels) are compatible with unaltered pore function. Fluctuation-based analysis, on the other hand, is endowed with single molecule sensitivity and high spatiotemporal resolution under native conditions but are typically conducted across many microns within the cell, thus averaging the contribution of many pores at the same time. Worthy of note, high-speed atomic force microscopy (HS-AFM) has been recently proposed as a valuable innovative approach to the study of the spatiotemporal dynamics of the NPC transport barrier [75]. In fact, Sakiyama and co-workers were able to visualize the nanoscopic spatiotemporal dynamics of FG Nups inside *Xenopus laevis* oocyte NPCs at a timescale of about 100 ms and conclude that the highly flexible, dynamically fluctuating FG Nups of the central channel rapidly elongate and retract, but do not cohere into a tightly crosslinked meshwork [75]. Although informative and very promising, the HS-AFM approach still represents an average of pore dynamic behavior on a time scale (hundreds of milliseconds) far from that typical of ucleocytoplasmic transport in vivo through NPCs (milliseconds). To tackle this latter relevant timescale, Cardarelli and co-workers proposed to use a feedback-based fluorescence tracking method, previously used to track point-like particles [76], to compensate for local diffusion of the entire NPC in living, unperturbed cells (Fig. 1D) [77], [78]. The measurement is conducted by rapidly orbiting the laser spot around the object of interest (the pore in this case), with a temporal resolution in the millisecond time window and localization precision in the nanometer range. Thus, standard analytical tools (e.g. fluctuation analysis) can be potentially brought onto the moving reference system of a *single* NPC to probe the movement of *single* molecules with high accuracy [77], [78]. In principle, the correlations in space and time due to single molecules crossing the NPC can be probed by fluctuation analysis along the orbit.

As a proof of principle, Cardarelli et al. recently exploited feedback-based tracking of the pore position by using GFP-labelled Impβ1 as a dynamic marker of the NPC in live, unperturbed cells [77], [78]. A circular light envelope is formed around the NPC, while the center of mass of the NPC is maintained at the center of the orbit by the feedback routine. The combination of this approach with fluctuation analysis allowed monitoring the transport of single molecules across single pores with high spatiotemporal resolution. Transport of Importin-β1 was detected as being regulated in such a way as to produce a characteristic distribution of transit times spatially restricted to the pore, and function of the metabolic energy. Similarly, the nucleoporin Nup153 (recently implicated in the transport of Impβ) was found to be regulated so as to produce rapid and discrete exchange between two separate positions within the pore. Based on these results, it was argued that directed Nup153-mediated Importin motion might represent a key component of the overall selective-gating process within intact NPCs, as envisioned by the polymer-brush model of NPC function [16], [79]. Our data do not exclude alternative patterns of dynamic interactions between the cargo and the NPC; however, they suggest that directed transport rather than passive diffusion may play a relevant role in the regulation of molecular transport across the NPC.

In summary, the orbital tracking method proved the potentiality to extract information on single-molecule events in a moving, nanoscopic reference system, in presence of many similarly-labelled molecules, and under physiological conditions. As such, this approach holds the promise to become a valuable technological platform to address the lingering questions at the level of single pores.

## Figures and Tables

**Table 1:** Summary of the pros and cons of presented techniques.

Methods^a^	Single-molecule	Single-pore	T-res	S-res	Labels	Sample preparation	Skills
Perturbation-based	No	No	Seconds^c^	Sub-cellular scale^d^	FPs	Transfection	Non-experts
Localization-based	Yes	Yes	10^− 4^–10^− 3^ s	Nanoscale	Large labels (e.g. QD) or organic dyes	Permeabilization/electroporation/transfection	Experts
Fluctuation-based	Yes	No^b^	10^− 4^–10^− 3^ s	Diffraction	FPs	Transfection	Experts

a The three classes of methods are here described strictly in the context of their application to nucleocytoplasmic transport.

b Except in the case of line-scan FRAP, where time resolution of few milliseconds can be reached (see for instance Ref. [49] of the main text).

c Except in the case of FCS combination with orbital tracking, as discussed in the future directions.

d Example: the entire nucleus/cytoplasm.
